# A Case of Intercurrent Dengue and Probable Relapsing *Plasmodium vivax* Malaria in a Returned Traveler to India: Case Report and Literature Review

**DOI:** 10.3390/pathogens14100987

**Published:** 2025-09-30

**Authors:** Kumudhavalli Kavanoor Sridhar, Fahad Buskandar, Manreet Dhaliwal, Gordane V. Calloo, Andrea K. Boggild

**Affiliations:** 1 Division of Medical Microbiology, Department of Laboratory Medicine and Pathobiology, University of Toronto, Toronto, ON M5S 1A8, Canada; 2 Division of Infectious Disease, John Hopkins University, Baltimore, MD 21287, USA; 3 Division of Infectious Diseases, Department of Medicine, University of Toronto, Toronto, ON M5S 3H2, Canada; 4 Faculty of Arts and Science, University of Toronto, Toronto, ON M5S 3G3, Canada; 5 Tropical Disease Unit, Toronto General Hospital, Toronto, ON M5G 2C4, Canada; 6 Institute of Medical Science, Temerty Faculty of Medicine, University of Toronto, Toronto, ON M5S 3K3, Canada; 7 Office of Access and Outreach, Temerty Faculty of Medicine, University of Toronto, Toronto, ON M5S 1A8, Canada

**Keywords:** arboviruses, dengue fever, fever in the returned traveler, malaria, relapsing malaria

## Abstract

Dengue and malaria are common vector-borne tropical diseases and are associated with high morbidity and mortality. Co-infection of dengue and malaria is underestimated due to parsimonious diagnostic approaches once the diagnosis of either is made, particularly using point-of-care assays, such as rapid diagnostic tests (RDTs). We present a case of dengue and *Plasmodium vivax* co-infection in a returned traveler from an endemic region, in whom the epidemiology and clinical course are highly suggestive of dengue triggering a *P. vivax* relapse. The literature on the co-occurrence of dengue and malaria in travelers is reviewed, as is the state of knowledge surrounding dengue as a precipitant to relapsing malaria.

## 1. Introduction

Dengue and malaria are common tropical vector-borne infections that are transmitted by the day-biting *Aedes aegypti* and night-biting *Anopheles* spp. mosquitoes, respectively. Dengue and malaria share an overlapping epidemiologic distribution and may manifest as a similar clinical syndrome, which makes them difficult to differentiate on purely clinical grounds. With vector-range expansion, increasing population urbanization and rising levels of co-endemicity globally, the recognition that intercurrent dengue and malaria co-infection occurs has become more important. In this report, we describe a case of *Plasmodium vivax* (*P. vivax*) malaria diagnosed in the context of intercurrent dengue fever in a febrile returned traveler from India and Mexico, with epidemiology and clinical presentation strongly supporting that the latter triggered relapse of the former. We herein provide a review of the published literature to date around the topic of both arboviruses triggering relapsing malaria, as well as reported cases of co-infection.

## 2. Case Presentation

A 27-year-old man of Indian origin who resides in Canada took a 15-day trip to New Delhi, India, to visit his friends and relatives (VFR) during the month of December and remained well during his travel and return. Ten days after returning to Canada, he developed fatigue, myalgia, headache and generalized weakness. Two days later, symptoms worsened with fever and chills, whereupon he travelled to Cancun, Mexico, for 3 days for tourism, during which time he continued to feel unwell and experience daily fevers. After returning to Canada, he presented to the emergency department (ED) since his symptoms were persisting, with worsening of high fevers to 40.5 °C.

On assessment in the ED, he was noted to be afebrile (37.5 °C), with a heart rate of 88 beats per minute, blood pressure of 116/77 mmHg, respiratory rate of 17 breaths per minute and oxygen saturation of 98% on room air. Physical examination at the time was notable for mild tenderness in the left upper quadrant on palpation without signs of organomegaly, masses, or acute abdomen. The remainder of the clinical examination at the time was unremarkable.

Complete blood count (CBC) was notable for mild anemia, with hemoglobin of 127 g/L (reference 140–180 g/L), total white blood cell (WBC) count of 4.9 × 10^9^/L (reference 4.0–11 × 10^9^/L) with moderate lymphopenia of 0.3 × 10^9^/L (reference 1.5–4.0 × 10^9^/L) and profound thrombocytopenia of 66 × 10^9^/L (reference 150–400 × 10^9^/L). Hepatic transaminases and creatinine were normal, but total bilirubin was mildly elevated at 37 µmol/L (reference < 22 µmol/L).

Initial work up for malaria in the ED by rapid diagnostic test (RDT) demonstrated positive pan-*Plasmodium* aldolase and negative *P. falciparum*-specific histidine-rich protein-2 (HRP-2) antigen, which, coupled with his travel history, was most suggestive of a non-falciparum malaria, given few cases of HRP-2 mutant strains of *P. falciparum* from the Indian subcontinent. A thin blood smear demonstrated intraerythrocytic stages of *P. vivax* at a parasitemia level of 0.7%, and the patient was started on atovaquone–proguanil 1000/400 mg daily for 3 days with fatty meals. Other laboratory investigations drawn at the time in the ED included blood cultures and serologic tests for hepatitis A, hepatitis B and dengue virus, and the patient was discharged and given a diagnosis of fever and lymphopenia. While dengue-specific polymerase chain reaction (PCR) is available via our provincial reference laboratory, this test is almost never ordered via the ED due to the burden of required paperwork; as such, it was not ordered for this patient. NS1 antigen testing for dengue is not available either through local hospital laboratories or the provincial reference laboratory.

Five days after discharge from the ED, he was seen in our unit for a comprehensive vivax malaria consultation and for follow-up. During presentation to our unit, he was feeling better already and his fever had fully resolved. He completed the 3 days of atovaquone–proguanil as prescribed and tolerated it well. The results of his workup from the ED revealed reference laboratory confirmation that his malaria was caused by the *P. vivax* strain. As such, he was started on primaquine phosphate 30 mg PO daily for 14 days for a radical cure, after confirming a normal G6PD level of 8.3 U/g Hb (reference 7.0–18.0 U/g Hb). His blood smear was repeated to document clearance post treatment, and parasitemia was reported as 0%. A repeat CBC demonstrated resolution of his lymphopenia (2.1 × 10^9^/L) and thrombocytopenia (232 × 10^9^/L).

Blood for dengue serologic testing was collected during the patient’s first visit to the ED (drawn on day 8 of fever) and was processed by the Public Health Ontario Laboratory; the test result was reported as dengue IgM and IgG reactive using a commercial enzyme immunoassay (EIA; Euroimmun Medical Diagnostics Canada Inc., Mississauga, ON, Canada). This result confirmed the occurrence of an acute dengue infection in our patient also experiencing acute malaria (a co-infection). By the time he was seen for specialized care in our tropical medicine unit, he was well, and as such, no further dengue-specific diagnostics (such as PCR) were undertaken. He remained clinically stable without bleeding manifestations throughout the entire clinic illness. The remainder of his infectious workup was negative.

At his second follow-up assessment in our unit the week after his first, the patient was well, appearing to tolerate primaquine. He denied any symptoms including fever, arthralgia or weakness. Repeat parasitemia was 0%. In light of his dengue IgM and IgG positivity on day 8 from symptom onset, this could represent either a secondary dengue infection or primary infection, as IgG can seroconvert after 7 days of illness, and we do not have results of any previous antibody titers that could help differentiate between primary and secondary infection.

He was seen again during a follow-up at our clinic after 6 weeks from symptom onset and continued to remain clinically well. Given the lack of quantitative antibody testing availability, or the ability to discriminate between dengue serotypes serologically using our provincial assays, his serology was not repeated during convalescence. His CBC at the time of follow-up was within the normal range, and his routine repeat day 28 parasitemia level was 0%. He was educated about mosquito protective precautions including use of DEET- or icaridin-based repellents as well as permethrin-impregnated clothing and nets for his next travel to dengue and malaria endemic areas. He was encouraged to seek pre-travel care for malaria prophylaxis prior to his next trip and to inquire then about the status of licensure of a dengue vaccine in Canada. While several dengue vaccines are licensed and marketed in countries around the world, Canada has no such approved preventive intervention at this time.

## 3. Discussion

This case adds to the growing literature of intercurrent infection with dengue and *P. vivax* in travelers visiting the tropics. Such cases have been described in different regions around the world, including India, Pakistan and South America [1,2]. Intercurrent infection of dengue and malaria due to species such as *P. falciparum* and *P. ovale* was thought to be rare; however, as more attention is being given to the possibility of intercurrent infection given increasingly overlapping endemicities, the incidence of such co-occurrence found to range between 7.1% to 23% of patients initially diagnosed with dengue [1]. This range in incidence depends on factors like geography and the presence of vectors for both pathogens.

Table 1 provides a review and summary of cases of *P. vivax* and dengue co-infections reported in the literature [1,3,4,5,6,7,8,9,10,11,12]. According to the published literature, most such cases were from endemic countries or following travel to endemic regions, and the clinical diagnosis of dengue was mostly delayed due to early positive malaria rapid tests (which yield point-of-care results), which happened in our case as well [6,7,13,14,15].

There have been mixed findings regarding the risk of severe illness in concurrent dengue and malaria infections. A retrospective study conducted by Epelboin and colleagues from French Guiana and by Magalhaes and colleagues from Brazil in 2012 found that intercurrent infection may carry a risk of severe symptoms with severe anemia and thrombocytopenia compared to infection with either pathogen alone [2,3]. Conversely, a retrospective study from India by Mohabtara and colleagues in 2012 demonstrated the opposite in that there was decreased risk of severe malaria compared to malaria-only cases and fewer bleeding manifestations compared to dengue-only cases [1].

According to the published literature, moderate-to-severe thrombocytopenia along with leukopenia, bleeding manifestations of any kind and a low malaria parasitemia level in a malaria patient born and raised in an endemic area should increase the suspicion of dengue intercurrence [1,2]. In our case, the patient had a benign clinical course with moderate thrombocytopenia and lymphopenia and a low malaria parasitemia level (0.7%). He denied known history of *P. vivax* malaria in the past.

Like *P. ovale*, *P. vivax* is one of the malaria species that portends risk of relapse given its ability to form hypnozoites in the liver. Another theory for the cause of both pathogens being detected is concurrent rather than intercurrent infection, where *P. vivax* relapse is triggered following acquisition and manifestation of dengue virus. There has also been a hypothesis proposed that a bite from a mosquito that is not infected with the malaria parasite could trigger activation of *P. vivax* hypnozoites [13].

The probability and frequency of relapse of *P. vivax* highly depends on the geographic origin of the strain, which can overlap with the distribution of the *Aedes* dengue vectors. The Indian subcontinent is unique, with a high prevalence of tropical strains of *P. vivax* over the southern region. This strain is characterized by short incubation (i.e., time from mosquito bite to the primary blood-stage infection [erythrocytic stages]) and short latency (i.e., the time from the primary malarial attack to relapse) of approximately 1–6 months. However, the northern-region strains exhibit longer latency and less frequent relapse [6,15]. In addition, New Delhi, the northern region to which our patient traveled, is also a hyperendemic region of dengue [16]. Although we lack access to diagnostic tests that could accurately discriminate amongst the four dengue serotypes, based on this patient’s travel history, it is quite likely that he was infected with DENV-2 [17,18]. Based on the onset of symptoms following his return from India and prior to departure for Cancun, we do not believe that the trip to Mexico was causally related to his illness in any way.

Our patient’s recent travel to an endemic region (i.e., India) for both dengue and long-latency *P. vivax* malaria strains makes it challenging to distinguish whether the *P. vivax* infection was a primary infection or relapse triggered by dengue. However, on balance, given his epidemiology, which included moderate thrombocytopenia and deep lymphopenia at presentation, it is more likely that his newly acquired dengue triggered a relapse of *P. vivax*. Although he denied prior history of malaria, the initial infection may have been silent and therefore immemorable. Further corroborating the likelihood of *P. vivax* relapse rather than primary infection is the typically long incubation period of vivax malaria, where >60% of travelers with *P. vivax* do not manifest any symptoms at all until >2 months after leaving the endemic area [19]. Our patient, on the other hand, was only in India for 15 days, and then manifested symptoms within 10 days of return, so while it is theoretically possible to acquire primary *P. vivax* infection on such an itinerary, his manifestation of symptoms within a month of his earliest possible exposure is highly atypical.

## 4. Conclusions

An intercurrent infection of dengue and malaria might be more common than previously estimated, particularly as vector ranges expand with global warming and climatologic events permissive to mosquito generation. The severity of the intercurrent infection can be mitigated by early diagnosis and treatment of the malaria component while supportive measures are instituted for dengue. Patients presenting with either infection acquired from areas endemic for both coupled with the trifecta of a low parasitemia level, moderate-to-severe thrombocytopenia and leukopenia, and bleeding manifestations support the diagnosis of intercurrent infection, which should be excluded promptly using rapid dengue diagnostics such as NS1 antigen detection and/or PCR rather than just serologic diagnosis.

## Figures and Tables

**Table 1:** Cases of dengue and *P. vivax* co-detection reported in the literature.

Publication/ Year/ Country/PMID	No. of Concurrent *P. vivax* and Dengue Cases	Clinical Information	Diagnosis	Outcome	Comments
Our case Canada	1	Fever, chills, myalgia and frontal headache	Malaria: Peripheral blood smear and RDT Dengue: EIA	Survived without complications	VFR India
Kariyawasam et al., 2019 Canada PMID 31051263 [6]	1	Fever, chills, sweats, headache and epigastric discomfort	Malaria: Peripheral blood smear and RDT Dengue: EIA and PCR	Survived without complications	VFR India
Tazeen et al., 2017 India PMID 28910810 [9]	1	Fever	Malaria: Peripheral blood smear Dengue: PCR	Survived without complications	3-year-old child with triple infection: *P. vivax*, dengue and chikungunya
Rao et al., 2016 India PMID 26653975 [4]	10	Fever	Malaria: Peripheral blood smear and RDT Dengue: EIA (IgM), NS1 antigen, RDT and PCR	Survived without complications	Retrospective study January–December 2013
Magalhaes et al., 2014 Brazil PMID 25340346 [11]	40	Fever in the past 10 days	Malaria: Peripheral blood smear and PCR Dengue: EIA (IgM, NS1 antigen) and PCR	Most had hepatic injury as complications but recovered	Cross sectional 2009–2011 (in 2012 from 11 patients) [2]
Mushtaq et al., 2013 India PMID 23606854 [12]	1	Fever, chills, myalgia and headache	Malaria: Peripheral blood smear and RDT Dengue: EIA	Survived without complications	*P. vivax*/*P. falciparum*/Dengue co-infection
Mohapatra et al., 2012 India PMID 23428528 [1]	3	Fever <7 days	Malaria: Peripheral blood smear Dengue: IgM and NS1 antigen by RDT	Survived without complications	Prospective observational study (June–September 2011); one case had mixed *P. vivax* and *P. falciparum*
Epelboin et al., 2012 French Guiana PMID 22549018 [3]	73	Fever	Malaria: Peripheral blood smear Dengue: IgM and IgA EIA, NS1 antigen by RDT and PCR	Survived without complications	Retrospective study 2004–2007
Santana et al., 2010 Brazil PMID 21085859 [10]	2	Fever	Malaria: Peripheral blood smear Dengue: PCR	Survived without complications	From patients with fever living in four cities, 111 clinical samples tested for arbovirus
Abbasi et al., 2009 Pakistan PMID 19149976 [7]	25	Fever in the past 10 days	Malaria: Peripheral blood smear Dengue: EIA	Survived without complications	September 2007–January 2008 from Karachi
Kaushik et al., 2007 India PMID 17568646 [5]	1	Recurrence of fever following conventional antimalarial treatment and symptomatic dengue treatment; *P. vivax* was overlooked	Rechecked the blood smear for *P. vivax*	Survived without complications	*P. vivax*/*P. falciparum*/Dengue co-infection
Thangaratham et al., 2006 India PMID 16785712 [8]	1	Fever, chills, rigor, cough and hematuria	Malaria: Peripheral blood smear Dengue: EIA IgM, IgG	Survived without complications	First case report

Abbreviations: EIA, enzyme immunoassay; IgG, immunoglobulin G; IgM, immunoglobulin M; NS1, dengue non-structural protein 1; PCR, polymerase chain reaction; RDT, rapid diagnostic test; VFR, visiting friends and relatives.

## Data Availability

All available data are contained in this report.
